# The flora phenotype ontology (FLOPO): tool for integrating morphological traits and phenotypes of vascular plants

**DOI:** 10.1186/s13326-016-0107-8

**Published:** 2016-11-14

**Authors:** Robert Hoehndorf, Mona Alshahrani, Georgios V. Gkoutos, George Gosline, Quentin Groom, Thomas Hamann, Jens Kattge, Sylvia Mota de Oliveira, Marco Schmidt, Soraya Sierra, Erik Smets, Rutger A. Vos, Claus Weiland

**Affiliations:** 1 Computational Bioscience Research Center (CBRC), King Abdullah University of Science and Technology, 4700 KAUST, Thuwal, 23955–6900 Kingdom of Saudi Arabia; 2 Computer, Electrical and Mathematical Sciences & Engineering Division (CEMSE), King Abdullah University of Science and Technology, 4700 KAUST, Thuwal, 23955–6900 Kingdom of Saudi Arabia; 3 College of Medical and Dental Sciences, Institute of Cancer and Genomic Sciences, Centre for Computational Biology, University of Birmingham, Birmingham, B15 2TT United Kingdom; 4 Institute of Translational Medicine, University Hospitals Birmingham, NHS Foundation Trust, Birmingham, B15 2TT United Kingdom; 5 Institute of Biological, Environmental and Rural Sciences, Aberystwyth University, Aberystwyth, SY23 2AX United Kingdom; 6 Royal Botanical Gardens, Kew, Richmond, Surrey, TW9 3AB United Kingdom; 7 Botanic Garden Meise, Nieuwelaan 38, Meise, 1860 Belgium; 8 Naturalis Biodiversity Center, P.O. Box 9517, Leiden, 2300 RA The Netherlands; 9 Senckenberg Biodiversity and Climate Research Centre (BiK-F), Senckenberganlage 25, Frankfurt am Main, 60325 Germany; 10 Max Planck Institute for Biogeochemistry, Hans Knoell Str. 10, Jena, 07745 Germany; 11 German Centre for Integrative Biodiversity Research (iDiv) Halle-Jena-Leipzig, Deutscher Platz 5e, Leipzig, 04103 Germany

**Keywords:** Phenotype, Biodiversity, Flora, Botany, Morphological traits

## Abstract

**Background:**

The systematic analysis of a large number of comparable plant trait data can support investigations into phylogenetics and ecological adaptation, with broad applications in evolutionary biology, agriculture, conservation, and the functioning of ecosystems. Floras, i.e., books collecting the information on all known plant species found within a region, are a potentially rich source of such plant trait data. Floras describe plant traits with a focus on morphology and other traits relevant for species identification in addition to other characteristics of plant species, such as ecological affinities, distribution, economic value, health applications, traditional uses, and so on. However, a key limitation in systematically analyzing information in Floras is the lack of a standardized vocabulary for the described traits as well as the difficulties in extracting structured information from free text.

**Results:**

We have developed the Flora Phenotype Ontology (FLOPO), an ontology for describing traits of plant species found in Floras. We used the Plant Ontology (PO) and the Phenotype And Trait Ontology (PATO) to extract entity-quality relationships from digitized taxon descriptions in Floras, and used a formal ontological approach based on phenotype description patterns and automated reasoning to generate the FLOPO. The resulting ontology consists of 25,407 classes and is based on the PO and PATO. The classified ontology closely follows the structure of Plant Ontology in that the primary axis of classification is the observed plant anatomical structure, and more specific traits are then classified based on parthood and subclass relations between anatomical structures as well as subclass relations between phenotypic qualities.

**Conclusions:**

The FLOPO is primarily intended as a framework based on which plant traits can be integrated computationally across all species and higher taxa of flowering plants. Importantly, it is not intended to replace established vocabularies or ontologies, but rather serve as an overarching framework based on which different application- and domain-specific ontologies, thesauri and vocabularies of phenotypes observed in flowering plants can be integrated.

**Electronic supplementary material:**

The online version of this article (doi:10.1186/s13326-016-0107-8) contains supplementary material, which is available to authorized users.

## Background

For hundreds of years, information on plant species found across the world has been collected in Floras, taxonomic monographs and annotations to collection material. Floras are books collecting the information on all known plant species found within a region. They describe plant traits with a focus on morphology and other traits relevant for species identification in addition to other characteristics of plant species, such as ecological affinities, distribution, economic value, health applications, traditional uses, and so on. Floras not only allow identification of plants found within a region, but also provide a large knowledge base of the phenotypic diversity found within ecosystems. The systematic analysis of such large-scale trait data can support investigations into phylogenetics and ecological adaptation, with broad applications in evolutionary biology, conservation, and the functioning of ecosystems. Moreover, the provision of trait data enables integrated knowledge discovery for agriculture (i.e. plant breeding) and phytomedicine. In particular many medicinal plants are not as comprehensively characterized as food crops or model plant systems. A comprehensive overview of the Floras available at the global level is given by [1]. A key limitation in systematically analyzing information in Floras is the lack of a standardized vocabulary for the described traits as well as the difficulties in extracting structured information from free text.

To facilitate integration and analysis of the information contained in Floras, we have developed the Flora Phenotype Ontology (FLOPO), an ontology for describing traits of plant species found in Floras. Ontologies provide formal, machine-readable definitions of the vocabulary used within a knowledge domain [2, 3]. The FLOPO builds on existing ontologies for morphological structures and phenotypic qualities, in particular the Plant Ontology (PO) [4] and the Phenotype And Trait Ontology (PATO) [5]. We have used these ontologies to extract entity-quality relationships from digitized taxon descriptions in Floras, and used a formal ontological approach based on phenotype description patterns [6] and automated reasoning to generate the FLOPO. Phenotype description patterns are formal statements in the Web Ontology Language (OWL) [7] that express the content of a phenotype description, i.e., the features of an organism when it has a particular phenotype.

The FLOPO allows integration of qualitative trait data from different sources, including text-based descriptions of phenotypes, such as those found in Floras and monographs, image-based representations of plant traits such as those found in photos and specimen scans (e.g., information stored in herbaria), as well as information about traits and phenotypes in trait databases such as TRY [8] or the Encyclopedia of Life’s TraitBank [9]. Through its links to established ontologies, it can also be used to link this data to data sources from other domains, such as genomics, macroecology or systems biology.

In our initial use case, our aims were to (1) identify the traits associated with taxa in Floras, (2) represent the traits in a semantic form amenable to computational analysis, (3) link the traits to standard vocabularies of plant morphology used in related areas of biological research, (4) and demonstrate that these traits can subsequently be integrated and compared with traits recorded in other databases. The FLOPO is freely available under a CC-0 license at http://purl.obolibrary.org/obo/flopo.owl.

## Methods

### Data sources

Building upon a collaborative prototype developed at the 2014 Biodiversity Data Enrichment Hackathon [10], an event similar to the popular BioHackathon series [11], we used several Floras (*Flora Malesiana* [12], *Flore du Gabon* [13, 14], *Flore d’Afrique Centrale* [15], *Flore du Congo Belge et du Ruanda-Urundi* [16], and a collection of Kew’s African Floras available at http://www.kew.org/science-conservation/research-data/science-directory/projects/e-floras, including the *Flora Zambesiaca*, *Flora of Tropical East Africa*, *Flora of West Tropical Africa*, *Flora of Tropical Africa*, *Flora Capensis* and the *Useful Plants of West Tropical Africa*). The Floras were available in digitized form, with most Floras written in English, and three in French (*Flore d’Afrique Centrale*, *Flore du Congo Belge et du Ruanda-Urundi*, and *Flore du Gabon*).

We assembled a vocabulary of plant morphological entities, attributes and attribute values. The terms for this vocabulary were taken from ontologies that are widely used in biological research: PO [17] for plant morphological entities, and PATO [5] for attributes and attribute values. Each ontology provides one or more English terms associated with one kind of plant entity (i.e., the labels and synonyms of classes in the ontologies). To identify the French terms associated with these entities, we used a dictionary provided by the Missouri Botanical Garden at http://www.mobot.org/mobot/glossary/ that was used by the project partners in the context of the FlorML project [18]. As result of this step, we obtained two dictionaries comprised of French and English terms for plant morphological entities, and attributes and attribute values.

### Text processing

Floras are available in different formats, including the structured XML-based format FlorML [18] as well as free text in taxonomic databases. In each Flora, we identified taxon names and identifiers together with complete (textual) taxon descriptions. We then processed the text using natural language processing (NLP) tools provided by the Apache Lucene [19] standard analyzer (basic stemming, stopword removal), applied a sentence identification method to tokenize the text into sentences (using the OpenNLP toolkit https://opennlp.apache.org/) and stored the resulting sentences together with their taxon names and identifiers in a fulltext index using the Apache Lucene framework.

We then applied the same stemming and stopword removal steps on the labels and synonyms of the ontology classes, and used Lucene to query the full text index for taxa descriptions in which sentences contain both a label or synonym of a quality (from PATO) and a label or synonym from a morphological entity (from PO). When querying French Floras, we first performed a dictionary-based translation of the labels, then applied the same pre-processing as applied to the textual taxa descriptions and performed the same query. Finally, we used the Stanford parser [20] to identify whether the quality term stands in an attributive relationship to the entity term.

As a result, we identified Entity-Quality pairs [5] in which entity-terms refer to plant morphological entities (from the Plant Ontology), and quality-terms to attributes or attribute values (from PATO). For example, from a sentence “The flowers are red” we identify the entity-quality pair (*Flower*, *Red*), where *Flower* is taken from the Plant Ontology (PO:0009046), and *Red* is taken from PATO (PATO:0000322). More complex relationships, such as connectivity between two morphological structures, are expressed as ternary relations in PATO (requiring the two connected entities and an additional instance of a relational quality as arguments), and we ignore them in our analysis; instead, we introduced placeholders which state that each structure is related to *something* without providing information on the second entity.

To filter the results, we used lexical parsing to determine whether the sentence expresses an attributive relationship between the quality and the entity we identified. For example, in the sentence “The flowers are red with yellow stamens.”, an attributive relationship exists between *Flower* and *Red* as well as *Yellow* and *Stamen*.

As a result of this text processing pipeline, we obtained a set of 502,693 PATO-based entity-quality descriptions of traits found in the Floras we analyzed. The entity-quality based descriptions consist of 20,584 distinct combinations of morphological structures from PO and qualities from PATO, using 287 distinct plant morphological structures and 545 distinct qualities, and are associated with 26,104 taxa.

### Ontology generation and automated reasoning

To generate the FLOPO, we use the extracted information in phenotype definition patterns [6], i.e., OWL axiom patterns for defining classes of phenotypes. We mainly generate three types of classes which we fully define in OWL: first, we create grouping classes representing the phenotypes of a plant structure or any of its parts (e.g., *flower phenotype*); second, we create classes for traits (or characters) of plant structures (e.g., *flower color*); finally, we create classes for the values of traits (or character states) of plant structures (e.g., *flower red*).

Using OWL, we generate the following classes and axioms for each entity-quality pair (*E*,*Q*):

- ’E phenotype’ EquivalentTo: has-part some ((part-of some E) and has-quality some quality)
- ’E Q’ EquivalentTo: has-part some (E and has-quality some Q)
- If *Q* is in the values subset of PATO, we identify the most specific superclass *T* of *Q* that is in PATO’s attribute subset, and generate the axiom ’E T’ EquivalentTo: has-part some (E and has-quality some T).

For example, for the entity quality pair (*f* *l* *o* *w* *e* *r*,*r* *e* *d*), we generate

- ’flower phenotype’ EquivalentTo: has-part some ((part-of some flower) and has-quality some quality)
- ’flower red’ EquivalentTo: has-part some (flower and has-quality some red)
- ’flower color’ EquivalentTo: has-part some (flower and has-quality some color)

The intuition behind our axiom patterns is that they always define a phenotype with respect to what must be true for a whole organism if the phenotype is present. For this purpose, we prefix every axiom with a has-part some restriction.

The use of this prefix pattern allows combining simple phenotypes (expressed through a single entity-quality pair) into complex phenotypes (requiring combinations of entity-quality pairs) through a simple intersection; for example, to describe the complex phenotype of having both red flowers and yellow stamens, the *flower red* and *stamen yellow* phenotypes would be intersected to form the complex phenotype of a whole organism having two parts, flowers that are red and anthers that are yellow. Without such a prefix, phenotypes could not easily be combined in such a way since *flower* and *anthers* are disjoint morphological entities, and *red* and *yellow* are disjoint qualities.

The parthood relation is used in another pattern to group traits by plant morphological structure as well as all parts of that morphological structure. In particular, the part-of relation (the inverse of the has-part relation) is both reflexive and transitive, and therefore subclasses of part-of some X include *X* as well as all classes with instances that necessarily are a part of some *X*. For example, subclasses of part-of some flower include, among others, *flower*, *petal*, and *androecium*, and using the parthood relation in the definition of the phenotype classes will lead to *petal phenotype*, *androecium phenotype*, etc., to become subclasses of *flower phenotype*. We do not use this pattern on the level of traits such as *flower color* as the traits of the parts will be different from the trait of the flower (e.g., the flower may be red while its stamens are yellow).

To distinguish between traits and their values, we use the distinction between attributes and attribute values in PATO in which classes are tagged through an annotation property either as *attribute* or *value*. When using the OBO Flatfile Format [21], these distinctions are expressed as the attribute slim and value slim of PATO.

Following the generation of the axioms for FLOPO based on the axiom patterns, we added one further axiom to the resulting ontology to remove impossible combinations of entity and quality, in particular those in which a morphological structure in PO is asserted to have a quality that can only be the quality of processes:



The ontology was generated using a Groovy script based on the OWL API [22] and the Elk reasoner [23]. Source code for processing Floras and generating the ontology is freely available (under a BSD-style license) at https://github.com/flora-phenotype-ontology/flopoontology.

## Results

### Data-driven generation of the Flora Phenotype Ontology

**Fig. 1:**
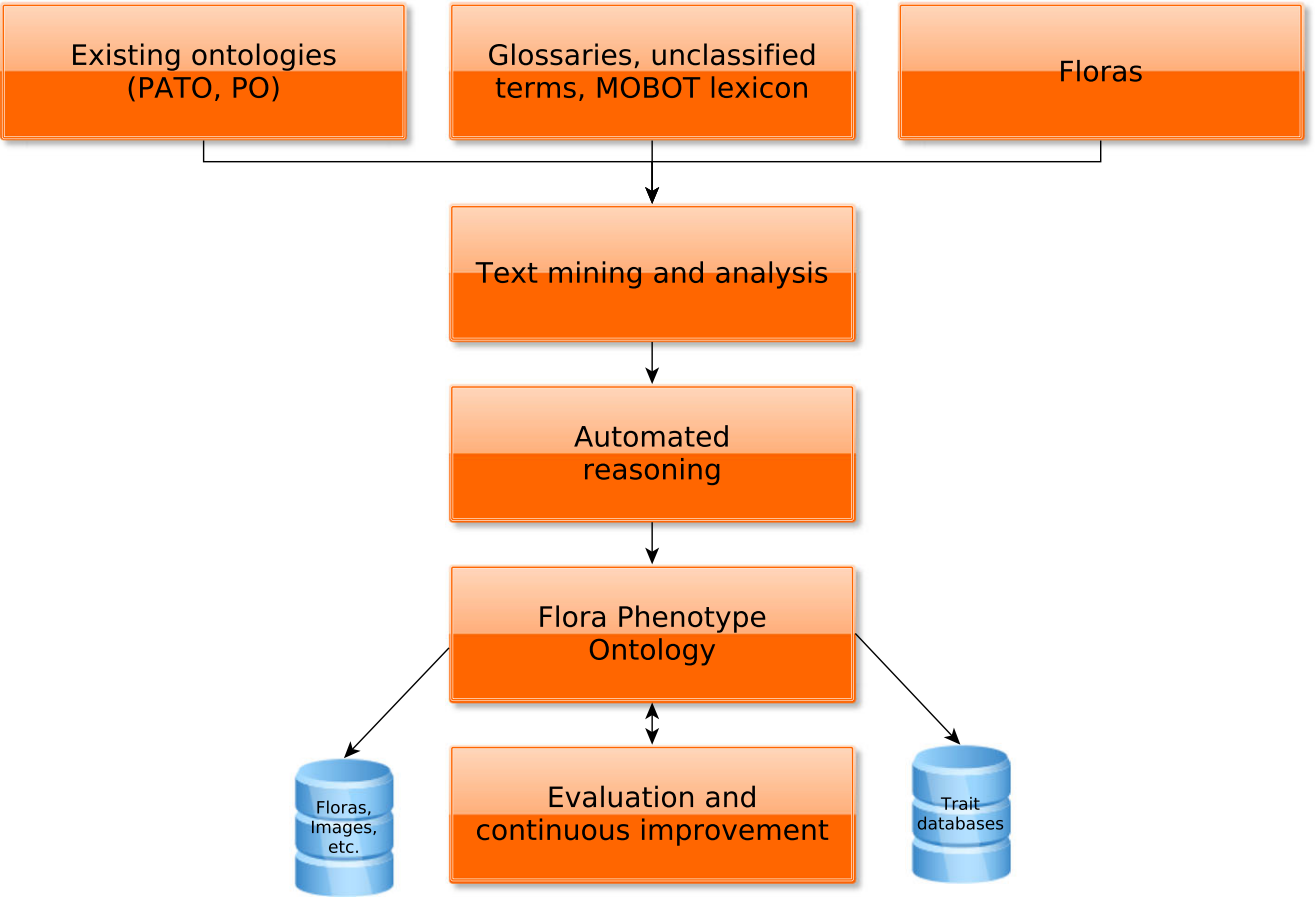
Overview over the main workflow in generating the FLOPO. After generation of the FLOPO, it can then be used to access plant trait and phenotype data in different databases

For creating the Flora Phenotype Ontology we used as primary use case the traits and phenotypes described in the Floras listed in the Methods section. Figure 1 provides an overview of our workflow. Using the Entity-Quality pairs extracted from the Floras, we developed a data-driven approach to generate a prototype of an ontology that would likely be capable of characterizing a large number of the traits observed in our study. In each Entity-Quality pair, the ‘entity’ term directly maps to a morphological entity (in the Plant Ontology), and the ‘quality’ term maps to an attribute or value (in the PATO ontology). We aimed to exploit the background knowledge in these ontologies together with an automated reasoner to generate an ontology in which each class characterizes a trait and is associated with at least one taxon in one of the Floras we processed. Specifically, we aimed to exploit the information about parthood relations between morphological structures and biological processes, and the subclass relations between qualities, morphological parts and physiological processes to generate the ontology [6]. We used a pattern-based approach in which we create axiom patterns that combine information obtained through our NLP-based approach with information in the referenced ontologies. Through the axiom patterns, we achieve:

- structural organization based on anatomical parthood (e.g., a *petal phenotype* should become a subclass of the *flower phenotype* based on *petal* being a part of *flower*),
- separation of types of trait for each morphological structure (e.g., a *flower color* should be separate from the *flower shape*), but both should be more closely related to each other than to *root color* as both are *flower* traits,
- separation and structural organization of attributes and values (e..g, *flower red* should become a subclass of *flower color*), and
- semantic interoperability with existing ontologies in the plant domain, including the Plant Ontology and Trait Ontology.

### Flora Phenotype Ontology

The Flora Phenotype Ontology (FLOPO), available at https://purl.obolibrary.org/obo/flopo.owl, is the result of classifying the axioms generated from our text-mining pipeline together with the PATO and PO ontologies. Classification of an ontology is a reasoning task in which the axioms within the ontology are used to determine the most specific sub- and super-class for each class in the ontology. As all generated axioms are in the OWL EL profile [24], we used the Elk reasoner [23] to perform the classification. The resulting ontology, the FLOPO, consists of 25,407 classes (24,076 classes unique to the FLOPO, in addition to the classes in PO and PATO). Each class is assigned a unique IRI in the namespace http://purl.obolibrary.org/obo/FLOPO_ followed by a unique numerical identifier. For example, the class *flower red* has the identifier FLOPO:0007599 when using FLOPO: to refer to the FLOPO namespace, i.e., FLOPO:0007599 will refer to the IRI http://purl.obolibrary.org/obo/FLOPO_0007599.

**Fig. 2:**
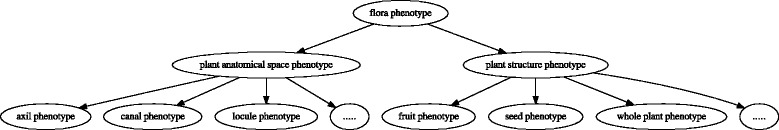
An overview of the top-level structure of the FLOPO

The classified ontology closely follows the structure of PO in that the primary axis of the classification shows the observed plant anatomical structure, while more specific traits are classified based on parthood and subclass relations between anatomical structures as well as subclass relations within PATO. Figure 2 shows the upper level of the FLOPO.

As most classes in the FLOPO are fully defined using axioms in OWL, it can be queried using either the labels of a class, the identifier of a class, or semantically using the axioms that are used to define the class. The latter kind of query is particularly useful when querying for classes that are not currently contained within the FLOPO. For example, the FLOPO does not currently contain a class for the *flower* being *deep pink*. Nevertheless, a semantic query using the entity-quality pair *flower* and *deep pink* and the axiom patterns we would use within the FLOPO (has-part some (flower and has-quality some ’deep pink’), it is possible to query for the equivalent or direct superclasses of that description which will return flower pink as the closest matching class.

Following the automatic generation of the FLOPO, we have also begun to manually add classes to FLOPO based on user requests and our own use cases. While we aim to fully define all classes in FLOPO, some classes cannot be defined without also extending other ontologies such as PO or PATO. FLOPO currently contains 198 manually created classes of which more than 50 % are fully defined while the others are restricted by subclass axioms alone.

### Evaluation: coverage of traits in Floras and plant databases

To test the coverage of traits in FLOPO, we manually annotated taxon descriptions from Floras and evaluated the correctness and coverage of traits in FLOPO. Correctness refers to the creation of nonsensical classes generated by the automated analysis, while coverage (i.e., recall) refers to the number of characters in plant descriptions that have a corresponding FLOPO class.

We have not performed a quantitative assessment of how many of the classes in FLOPO do not make sense but did a qualitative analysis instead. Classes such as *xylem vessel member tomentose*, *peduncle female*, and *lower glume subacute* are obviously artifacts of the automated generation and constitute a significant number of classes in FLOPO. We distinguish two main sources of these artifacts:

- The parsing of the descriptions failed to correctly associate entities with attributes. Parsing of descriptions is difficult, and entities and qualities in the same sentences may incorrectly be identified as entity-quality pairs.
- Some labels of qualities in PATO can be used in another context to refer to completely different qualities. For example, *acute* in PATO is a quality of processes, used to characterize for example diseases such as *acute malaria*, while the term “acute” in a plant description usually refers to an angle. These qualities are then propagated up the hierarchy (due to inheritance in PATO) and yield further non-sensical classes (such as *leaf intensity*).

In response to our evaluation, we have manually deprecated several classes and added an axiom to prevent the use of any process qualities in FLOPO. Currently, 564 classes in FLOPO have been deprecated for this reason. While nonsensical classes clutter the ontology with useless classes, they do not prevent the use of the remaining classes in standardizing the description of traits.

To evaluate coverage of FLOPO, we have performed a rigorous application of the ontology to eight plant descriptions from several Floras and within a number of taxonomic groups. The detailed results can be found in the Additional files 1 and 2. We identified between 40 and 85 characters for each taxon, and the coverage of characters in FLOPO ranged from 48 to 70 %. Simple characters such as *stem diameter* are well represented in FLOPO. More complex characters, however, are often lacking, although some complex characters such as *petiole margin undulate* (often a useful character for identification) are present.

The largest number of missing classes in FLOPO are due to qualities missing in PATO. Examples of these include *caulescent*, *chartaceous*, and *axillary* (full list provided as Additional file 3). While *truncate* is present in PATO, we did not match *truncated* in our text processing method. Furthermore, some PO classes were also missed due to missing labels or synonyms and our use of exact matching in text processing. For example, *ovule* was not matched because it corresponds to the class *plant ovule* in PO which has no synonym “ovule”. Any plant organ class missing in PO leads to an absence of FLOPO classes for that organ. Additionally, some combinations of PO and PATO are not identified, sometimes due to the lack of comparative classes (or synonyms) in PATO such as *unequal* or *longer than*.

To further evaluate the coverage of FLOPO, we have used independent trait data from “African Plants – a photo guide” [25] database, an expert-based tool using trait data for identification purposes. The trait *life form* and quantitative traits such as the number of petals, that do not fit with the entity-quality terms in FLOPO, have been excluded beforehand. Out of 80,887 taxon-trait combinations, 44,200 (55 %) could be matched to FLOPO classes. Out of 88 traits that were used in the African Plants database, 31 were already present in FLOPO and 57 were manually created in FLOPO following this evaluation.

### The link to genetics: integrating wild-type and model organism phenotypes

Phenotypes are not only collected in a natural context, but also in the context of model organisms [26]. In many cases, model organism databases collect *abnormal* phenotypes [26]. These differ from phenotypes observed in a biodiversity context in the fact that they represent differences to a control group. For example, while a *flower red* phenotype in a biodiversity context states that the members of a particular species, or an individual sample of that species, have red flowers, it may indicate in a model organism context that, based on some experimental conditions such as a gene knockout or environmental alteration, the flowers of the organism are red under the experimental conditions while the control group has differently colored flowers. These experiments can provide useful information on functional genetics by revealing the phenotypic effects associated with particular genes or revealing the mechanisms underlying environmental adaptation [26, 27].

While the FLOPO is primarily focused on describing the traits and phenotypes in wild-type plants, its classes can also be used to characterize divergent phenotypes as, for example, observed in functional genetics experiments. To test this assumption we used a dataset of formal phenotype descriptions recorded in mutant models of *Arabidopsis thaliana*, maize, barrel medic, rice, soybean, and tomato [28]. Out of 5,186 phenotype statements contained in the dataset that involve a plant anatomical entity, 315 directly match one of the classes in the FLOPO, while the others have superclasses in the FLOPO. The low number of directly matching classes may be a consequence of the different way in which the phenotypes are recorded; in a model organism context, phenotype descriptions include statements such as *whole plant increased size* or *seed inviable*, which are not recorded, or meaningful, without an explicit group to which phenotypes are compared. Nevertheless, these results show that FLOPO can be used to combine plant phenotype data from different databases and domains.

## Discussion

### Interoperability with plant trait vocabularies

The FLOPO is primarily intended as a framework based on which plant traits can be integrated computationally across all species and higher taxa of flowering plants. Importantly, while FLOPO can be used for annotation directly, it is not intended to replace established vocabularies or ontologies, but rather serve as an overarching framework based on which different application- and domain-specific ontologies, thesauri and vocabularies of phenotypes observed in flowering plants can be integrated. Using the axiom patterns we defined and used to generate the FLOPO, any ontology-based phenotype description using the entity/quality method can be directly integrated with the FLOPO, and appropriate equivalent classes, sub- and super-classes can be identified using automated reasoning (either using an automated reasoner directly or querying through public repositories such as AberOWL [29] which provide reasoning services for ontologies, including the FLOPO).

Additional terminological resources, such as the Plant Trait Thesaurus [30], the Crop Ontology [31], the Plant Trait Ontology [32], as well as general and application-specific plant-related thesauri, can be integrated and semantically enriched through mappings to the FLOPO. These mappings can either be established manually by domain experts or, in some cases, automatically through mapping of labels.

### Multi-modal data sources

We have primarily used a large corpus of plant taxa in Floras as a source for the FLOPO. However, an increasing number of automated methods is being developed to detect traits, phenotypes and species from multi-modal information sources including photographs [33], herbarium sheets [34, 35], microscopy images [36], or schematic drawings. The FLOPO can also be utilized to integrate data obtained from different sources and analysis approaches. To achieve this goal, analysis methods that detect morphological traits and phenotypes in plants would either output FLOPO classes directly, or the output of these methods would be mapped to FLOPO classes.

As different data sources and analytic approaches have different error rates and levels of confidence, data sources that integrate multi-modal information should provide different kinds of evidence and additional information, at least the data source (e.g., the collection of which it is a part), the type of data (e.g., whether it is textual data, or photographs), the protocol that was applied to obtain the data, the data extraction method (e.g., image analysis, text mining), and the environmental conditions under which the phenotype has been observed. Different ontologies and checklists have been developed to capture these aspects of scientific data collection. For example, the Provenance Ontology (PROV-O) [37] can be used to specify the data source and authoring information. The Biological Collections Ontology (BCO) [38] can be used to specify the plant specimens mentioned in the species treatments and thereby link to geography and species concepts. The Plant Experimental Assay Ontology (PEAO) (https://bitbucket.org/PlantExpAssay/ontology/raw/v0.1/PlantExperimentalAssayOntology.owl) can be used to specify the assays that were used to process both the original plants of which phenotypes were recorded and the protocols used to collect the data. The EDAM ontology [39] can be used to specify how the data was extracted, e.g., whether FLOPO classes were assigned manually or automatically, and if the latter, which methods were used to extract the information. A crucial component in any description of observed phenotypes is the combination of environmental conditions under which the phenotypes have been observed, and several ontologies have been established for this purpose. In particular, the Environment Ontology (EnvO) [40] covers environments in which organisms are found and can also provide relevant classes applicable to plant biodiversity. We have also attempted to annotate the Floras in our study with classes from EnvO. However, in contrast to plant morphology and phenotypes, in which we can filter lexical matches by the syntactic relations between the term referring to a morphological entity and the term referring to a quality, we find that environmental conditions are more difficult to identify precisely using purely lexical approaches. Especially in Floras, environmental descriptions may be context-specific and require prior knowledge of the area. Future research will include developing and applying dedicated environmental named entity recognition approaches [41], as well as using additional plant-specific ontologies such as the Plant Environment Ontology [4] to precisely identify and characterize environmental conditions.

### Automatic generation of phenotype ontologies and comparison

The initial draft of FLOPO was generated from literature using a pattern-based approach in order to maintain a balance between trait descriptions that are actually used to characterize plants and the totality of all descriptions that are possible when using the PO and PATO ontologies. The axiom patterns we use in FLOPO are motivated primarily by the aim to generate an ontology in which the basic underlying taxonomy follows the distinctions made in classifying morphological structures in plants and are comprehensible to domain experts using the ontology. However, the axioms we use to define traits and phenotypes are distinctly different from the axioms used in other phenotype ontologies [42], including the widely used Mammalian Phenotype Ontology [43] and Human Phenotype Ontology [44]. The classes we generate are also not explicitly declared to be subclasses of *quality* (from PATO), as in some other ontologies and applications [42, 45]; while we do not perform an explicit analysis regarding the ontological state of our classes, the intention is that our axioms provide a description of a whole organism and what must be true of it when having a particular phenotype. These can either be considered as qualities of a whole organism (and therefore a subclass of PATO’s *quality* class), or equivalently as subclasses of the whole organism (a material entity) [6].

The pattern-based approach we use is inspired by recent suggestions to go beyond the quality-centric approach of defining phenotypes, and instead explicitly characterize the configurations of the whole organism that has the phenotype, including the parts the organism has or lacks, the processes it participates in or not, the functions is has, and the qualities is has or lacks [6, 46, 47]. These approaches have the advantage of explicitly being able to utilize knowledge from anatomy or physiology ontologies [6, 46], and have successfully been applied to integrate a large number of phenotype ontologies [28, 48]. However, a difference in axiom patterns to other phenotype ontologies may increase the effort required in integrating these ontologies with FLOPO. Should it be required to treat the classes in FLOPO as subclasses of *quality*, all our axiom patterns can further be prefixed with inheres-in some in order to make them subclasses of *quality*. These changes can be applied automatically without changing any of the inferences we describe [6], or increasing the expressiveness of the language required to express the axioms (i.e., OWL 2 EL).

### Continuing development of the FLOPO and its annotations

We used largely an automated and data-driven process to generate the FLOPO. As a consequence, the generated FLOPO contains several artifacts that are a consequence of the text matching process. In particular, it contains traits that are not relevant or measured, such as *bark surface area*, and may lack traits that are difficult to identify through a lexical approach. Therefore, after our largely automatic approach, we have already started to manually improve both correctness and coverage of FLOPO, and we aim to continue the development of the FLOPO further with involvement of domain experts. For this purpose, we provide an issue tracker (at https://github.com/flora-phenotype-ontology/flopoontology/issues) in which FLOPO users can request changes, ask for new classes to be added, and actively contribute to the further development of the FLOPO.

One instance of a further manual evaluation of the FLOPO by domain experts is an ongoing study at Naturalis (involving TH, SMO and RV) to extract homologized traits and their values, i.e., “characters” and “character states” in the context of evolutionary comparative analysis, for the economically valuable tropical plant family *Piperaceae*. In this study, automatically extracted entities and respective qualities are scrutinized by botanists, and their fidelity to the entity-quality context in the source evaluated. This longer-term study will help to further improve FLOPO.

We also aim to develop semantic annotations of taxa with the FLOPO. Currently, we are using a custom text processing pipeline to extract entity-quality pairs with the primary aim of building a comprehensive ontology. However, there is an extensive body of research on analyzing traits and phenotype found in text; in particular the CharaParser [49, 50] has achieved high accuracy in extracting formalized character statements from Floras, and we intend to evaluate its use in the future. We plan to apply similar methods and make FLOPO-based annotations of taxa available using Semantic Web technologies, and link the taxa to their corresponding International Plant Names Index (IPNI) [51] identifiers to enable interoperability with databases of plant traits and phenotypes. IPNI provides a service for URNs (LSID), which we are currently evaluating among other services like Identifiers.org (URL based) [52] to publish the taxon annotations as Linked Open Data (see Fig. 1).

## Conclusions

We have developed the Flora Phenotype Ontology (FLOPO), an ontology of plant traits and phenotypes found in Floras and monographs. The FLOPO is an ongoing, community-driven project, and is intended both for data annotation and as a framework based on which plant traits can be integrated computationally across all species and higher taxa of flowering plants.The FLOPO is being used for annotation of traits, in particular within the African Plants Database [25], and in ongoing projects for the annotation and integration of plant trait data.

## Additional files

Additional file 1
**Annotation of Floras 1.** The file contains the manual annotation of *Salacia erecta*, *Cucumeropsis mannii*, *Oxalis*, and *Basella alba*. The file includes identified phenotypes and the mapping to FLOPO. It also highlights missing classes in PATO or PO. (XLSX 24 kb)

Additional file 2
**Annotation of Floras 2.** The file contains the manual annotation of *Salacia erecta*, *Andropogon chinensis*, *Oxalis*, and *Anisopappus chinensis*. The file includes identified phenotypes and the mapping to FLOPO. It also highlights missing classes in PATO or PO. (XLSX 36 kb)

Additional file 3
**Missing PATO classes.** A list of missing classes in the PATO ontology identified by our study. (TXT 4 kb)

Additional file 4
**Flora descriptions.** The original descriptions of *Salacia erecta*, *Andropogon chinensis*, *Oxalis*, and *Anisopappus chinensis* based on which the manual annotation was performed. (PDF 24 kb)

## Abbreviations

BCO: Biological collections ontology
EnvO: Environment ontology
FLOPO: Flora phenotype ontology
IPNI: International plant names index
LSID: Life science identifier
NLP: Natural language processing
OBO: Open biological and biomedical ontologies
OWL: Web ontology language
PATO: Phenotype and trait ontology
PEAO: Plant experimental assay ontology
PO: Plant ontology
PROV-O: provenance ontology
URN: Uniform resource name
