# Supplementary material for: The flora phenotype ontology (FLOPO): tool for integrating morphological traits and phenotypes of vascular plants
Source: J Biomed Semantics. 2016 Nov 14;7:65. doi: 10.1186/s13326-016-0107-8 (PMC5109718; doi:10.1186/s13326-016-0107-8)
Supplement: Additional file 4 — Flora descriptions. The original descriptions of Salacia erecta, Andropogon chinensis, Oxalis, and Anisopappus chinensis based on which the manual annotation was performed. (PDF 24 kb) [file 13326_2016_107_MOESM4_ESM.pdf]

Taxa evaluated by GG:

Flora	Taxon	Description
Flora Zambesiaca	Oxalis (genus) Oxalidaceae	Annual or perennial herbs, rarely trees or shrubs or shrublets. Leaves alternate, exstipulate (but sometimes with stipule-like expansions at the base of the petiole), digitately or pinnately 1-? -foliolate. Flowers in axillary cymes or in pseudumbels or solitary, actinomorphic or nearly so, bisexual, 5-merous, often heterostylous, sometimes cleistogamous and reduced. Sepals 5, free, imbricate (rarely valvate). Petals 5, contorted or imbricate, free or slightly connate at the base. Stamens 10 (15), 2 (3)-seriate; anthers versatile, 2-thecous; filaments ± connate at the base. Ovary superior, 5-locular; loculi 1-?-ovulate, with axile placentation; styles 5 (rarely 1), free; stigmas capitate, entire. Fruit a loculicidally dehiscent capsule (rarely baccate). Endosperm fleshy or absent.
Flora of Tropical East Africa	Salacia erecta -- Celastraceae	Glabrous shrub or liane to 15 m. in height. Latex threads absent. Stems 4-angled or with paired ridges, green or purplish, becoming terete, dark purplish grey, smooth, or rough with conspicuous raised lenticels. Leaves opposite, subopposite or alternate, petiolate; petiole 3–6 mm. long with conspicuously undulate margins; blade papery or subcoriaceous, usually glossy dark green above, paler beneath, usually elliptic, sometimes lanceolate, (2.5–)3.5–11(–15) cm. long, 1.7–4.5(–9) cm. wide, usually bluntly acuminate, rarely obtuse or retuse, cuneate to almost rounded at the base, with fine slightly prominent reticulate veins beneath; margin serrate to subentire. Inflorescence glabrous, consisting of few-flowered sessile or shortly pedunculate axillary fascicles; bracts ovate, ± 0.5–1 mm. long, fimbriate; pedicels 4–8(–10) mm. long at anthesis, articulated at the base. Buds ovoid or conical, 2–3 mm. long. Flowers greenish yellow (? sometimes orange), ± 7–9 mm. in diameter, sepals free or very shortly joined at the base, unequal, ovate to semicircular, 0.5–1 mm. long, rounded, fimbriate. Petals oblong, 3–4 mm. long, rounded, entire. Disc a fleshy convex pad ± 2–2.5 mm. in diameter. Stamens with slender flattened filaments 1.5–2 mm. long, widened at the base. Ovary trigonous or 3-lobed, with a conical style ± 0.5 mm. long; stigma punctiform; ovules (2–)4–6 per locule. Fruit orange, ± globose or oblong at maturity, often apiculate, 1–2.5(–3) cm. in diameter, smooth or sometimes warted, especially towards the apex

Flora of Tropical East Africa	Andropogon chinensis - Poaceae	Tufted perennial; culms 60–200 cm. high. Leaf-blades 10–40 cm. long, 1–8 mm. wide, glabrous or pubescent, ± glaucous, often with sheath-auricles up to 4 mm. long. Inflorescence of paired (rarely more) racemes exserted from linear spatheoles and gathered into a loose, sometimes scanty, false panicle; racemes 3–7(–10) cm. long, pilose to villous; internodes and pedicels cuneate, ciliate on both margins with hairs 1–3 mm. long. Sessile spikelet 5–8 mm. long, including a short callus; lower glume linear, deeply depressed between the dorsal keels, glabrous; upper glume with an awn (1–)4–12 mm. long; upper lemma bidentate, with an awn 2–3.5 cm. long, occasionally less. Pedicelled spikelet lanceolate to narrowly elliptic, 4–7 mm. long, pallid to purplish, glabrous to pilose, mostly 2-awned, the longer (2–)4–10 mm. long, the other much shorter or sometimes suppressed.
Flora of China	Anisopappus chinensis - Asteraceae	Herbs, annual. Rhizome stout, 5-12 mm in diam. Stems erect, 40-100 cm tall, 2-4(-6) mm in diam., simple or rarely caespitose, with slender stripes, subwoody at base, densely rusty pubescent or sparsely pubescent in lower part during anthesis. Cauline leaves ovate-lanceolate or narrowly oblong, 3-6 × 1-2 cm, papery, both surfaces slightly pubescent, more densely so along veins, base truncate or broadly cuneate, margin obtusely serrate, apex obtuse. Capitula solitary or several in terminal corymbs; peduncles densely pubescent. Involucre hemispheric, 6-10 mm; phyllaries in 3 series, narrowly lanceolate or broadly linear, 3-5 × ca. 1.5 mm, dorsally densely pubescent, margin membranous, apex obtuse. Paleae keeled, membranous, ca. 5 mm. Marginal florets female; corolla yellow; lamina obtriangular, ca. 6.5 × 2 mm, apex truncate, 3-dentate. Disk florets bisexual; corolla tubular, ca. 3 mm, with short triangular lobes. Achenes columnar, sparsely pubescent, apex truncate, slightly narrower to base, ca. 2 mm in female florets and ca. 1.5 mm in bisexual florets, with 4 ribs. Pappus grayish white, chaffy, of 4 or 5 bristles.
